# Single amino acid in V2 encoded by TYLCV is responsible for its self-interaction, aggregates and pathogenicity

**DOI:** 10.1038/s41598-018-21446-2

**Published:** 2018-02-23

**Authors:** Wenhao Zhao, Yinghua Ji, Shuhua Wu, Xiaofang Ma, Shuo Li, Feng Sun, Zhaobang Cheng, Yijun Zhou, Yongjian Fan

**Affiliations:** 1 Department of Plant Pathology, Nanjing Agricultural University, Nanjing, 210095 China; 2 Institute of Plant Protection, Jiangsu Academy of Agricultural Sciences, Key Lab of Food Quality and Safety of Jiangsu Province—State Key Laboratory Breeding Base, Nanjing, 210014 China

## Abstract

The V2 protein encoded by *Begomovirus* is essential for virus infection and is involved in multiple functions, such as virus movement and suppression of the host defence response. In this study, we reported that V2 encoded by the *Tomato yellow leaf curl virus* (TYLCV), which is one of the most devastating tomato-infecting begomoviruses, could interact with itself and a S71A mutation of V2 (V2^S71A^) abolished its self-interaction. Fluorescence results showed that V2 localized primarily in the cytoplasm and around the nucleus. Site-directed mutagenesis V2^S71A^ had the similar subcellular localization, but V2^S71A^ formed fewer large aggregates in the cytoplasm compared to wild-type V2, whereas the level of aggregates came to a similar after treatment with MG132, which indicates that the S71A mutation might affect 26S proteasome-mediated degradation of V2 aggregates. Meanwhile, heterologous expression of V2^S71A^ from a Potato virus X vector induced mild symptoms compared to wild-type V2, delay of virus infection associated with mild symptoms was observed in plants inoculated with TYLCV-S71A, which indicates that the amino acid on position 71 is also involved in the pathogenicity of V2. To the best of our knowledge, this report is the first to state that the S71A mutation of V2 encoded by TYLCV affects the self-interaction, aggregate formation and pathogenicity of V2.

## Introduction

*Tomato yellow leaf curl virus* (TYLCV) is a monopartite begomovirus of the family *Geminiviridae*, which is transmitted by whiteflies and causes one of the most devastating diseases in tomatoes worldwide^1–4^. TYLCV contains a single genome component with six open reading frames (ORFs) and an intergenic region (IR). Four ORFs (C1, C2, C3 and C4) are located on the complementary strand, and the other two ORFs (V1 and V2) are located on the viral strand^5^. Replication-associated protein (Rep) encoded by C1, transcriptional activator protein (TrAP) encoded by C2 and replication enhancer protein (REn) encoded by C3 are involved in viral replication. C4 is likely involved in disease symptoms and viral movement. V1 is a capsid protein (CP) proposed to facilitate viral movement and assembly^1,6–8^.

Numerous studies had shown that begomovirus V2 proteins have diverse function associated with viral movement^9^, symptoms induction^10^, hypersensitive response (HR)^11^ and suppression of gene silencing^12^. For TYLCV V2, earlier studies have showed that V2 is involved in viral movement^7,13–16^. However, Hak *et al*. recently reported that this protein is essential for TYLCV infection, even though it is not directly involved in viral replication or movement^17^. While Moshe *et al*. found that V2 could bind genomic ssDNA and is occasionally co-localized with CP in large nuclear inclusions, it is likely to be involved in the CP-mediated DNA transport between the nucleus and cytoplasm, which indicates that V2 has a role as a DNA shuttling protein^18^. V2 was also reported to function as a viral suppressor of post-transcriptional gene silencing (PTGS) through interacting with slSGS3 and disturbing transportation of the RNA-silencing signal^19,20^. Recent research revealed that V2 could also suppress transcriptional gene silencing (TGS)^21^ in addition to PTGS. Moreover, Bar-Ziv *et al*. found that V2 could regulate plant defences through interacting with the tomato CYP1 protein, which is a member of the family of papain-like cysteine proteases^22^. Amin *et al*. found that expression of V2 via a Potato virus X (PVX) vector could induce severe symptoms in plants, which indicates V2 is involved in pathogenicity as an important symptom determinant^23^.

Many plant and animal viruses induce the formation of insoluble aggregates/inclusion bodies inside infected cells. These structures usually contain viral and host proteins and can vary in location, size, content and biological function. For TYLCV, a recent report on CP protein showed that the progress of virus infection was accompanied by the formation of CP aggregates of increasing size, which might be involved in viral replication and assembly that finally affects the development of the viral disease^24^. The TYLCV V2 protein was also reported to demonstrate aggregates formation in plants, and the formation and 26S proteasome-mediated degradation of V2 aggregates were dependent on the integrity of the actin and microtubule cytoskeleton, which indicated that the cytoskeleton-dependent formation and growth of V2 aggregates play an important role in TYLCV infection^18^.

In many plant viruses, self-interaction of viral proteins is associated with their function. For examples, the P6 protein encoded by S6 of *Rice black streaked dwarf virus* (RBSDV) could interact with itself, which is associated with the formation of viroplasm-like structures to produce replication sites^25^. In *Begomovirus*, the self-interaction of Rep encoded by *Abutilon mosaic virus* (AMV) is involved in initiating the replication in the endonucleus^26^. The AL2 protein encoded by *Tomato golden mosaic virus* (TGMV) could interact with itself to form multimeric structures of AL2 in the nucleus and to activate transcription efficiently, whereas AL2 monomers present in the cytoplasm of infected cells, suppress local silencing by interacting with adenosine kinase ADK^27^. A recent study also found that the βC1 protein encoded by *Tomato yellow leaf curl China betasatellite* (TYLCCNB) lead to multimerisation by interacting with itself, which shows the multimers and self-interaction are related to begomovirus-like symptoms in *N. benthamiana*^28^.

Here, we show that the TYLCV V2 protein interacts with itself and that the amino acid on position 71 is crucial for the self-interaction. A substitution of serine for alanine at this position (V2^S71A^) abolished its self-interaction, induced less cytoplasmic large aggregates formation and milder symptoms compared to wild-type V2. Additionally, our data indicate that an S71A mutation of V2 affects 26S proteasome-mediated degradation, which in turn altered the dynamics of aggregates formation. In summary, our results reveal a correlation between the amino acid on position 71, self-interaction of V2, aggregates formation and pathogenicity, which may have been largely unexplored in favour of determining the function of V2.

## Results

### V2 encoded by TYLCV interacts with itself in a yeast two-hybrid system

V2 encoded by TYLCV is a suppressor of gene silencing and essential for virus infection. To investigate the viral factors encoded by TYLCV that interacted with V2 and clarify their possible functions, yeast two-hybrid experiments were performed and the self-interaction of V2 was identified.

Primers were designed to amplify the full-length coding sequence, and the full-length gene encoding V2 was cloned. Sequence analysis revealed that the V2 open reading frame (ORF) contains 351 nucleotides (nt) (GenBank accession number: GU111505).

**Figure 1:**
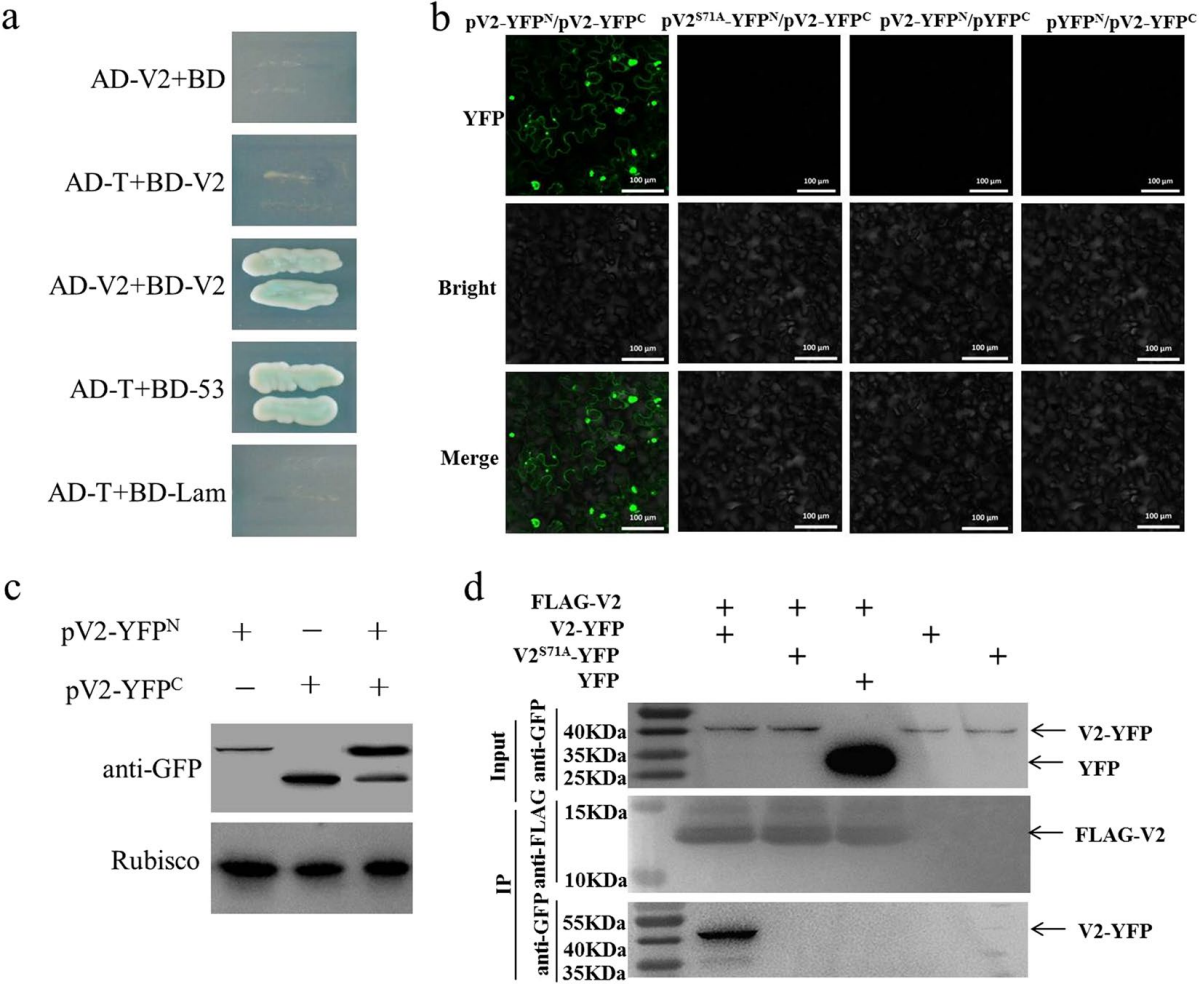
Identification of the interaction of V2 encoded by TYLCV with itself and mutants. (**a**) The self-interaction of TYLCV V2 in a yeast two-hybrid system. Co-transformed yeast strain AH109 grew on SD/-Ade/-His/-Leu/-Trp medium with X-α-gal. (**b**) BiFC visualization of the interaction between V2 and itself/mutants. Fluorescence was observed 48 hours post-infiltration. Bars: 100 μm. (**c**) Western blot analyses of BiFC construct combinations from the same experiments as in (**b**). All combinations were detected with anti-GFP polyclonal antibody (**d**) Co-Immunoprecipitation assay to show the *in vitro* self-interaction of V2 and the interaction of V2^S71A^ and V2.

Co-transformants of V2 cloned as a fusion with the GAL4 activation domain (AD-V2) and V2 as a fusion with the GAL4 DNA binding domain (BD-V2) were plated on different selective media to detect activation of the reporter genes HIS3 and ADE2. AD-V2 and BD-V2 were transformed into yeast (AH109) and the cells grew normally on SD/-Trp medium, which indicates that V2 non-specifically affected yeast-cell physiology. Yeast transformants containing AD-V2 and BD-V2 were able to grow and tended to appear blue on SD/-Ade/-His/-Leu/-Trp medium with X-α-gal, which was similar to the transformants containing AD-recT and BD-53 (positive control), whereas yeast transformants carrying AD-V2 with an empty vector (AD-V2 + BD) or BD-V2 with an empty vector (BD-V2 + AD) or AD-recT with BD-Lamp (negative control) were unable to grow on the synthetic medium lacking Ade, His, Leu and Trp (Fig. 1a).

### V2 interacts with itself in plants

The self-interaction of V2 was further investigated by bimolecular fluorescence complementation (BiFC) in agro-infiltrated *N. benthamiana* leaves. In this assay, V2 was fused to the N (YFPN) and C-terminal (YFPC) fragments of yellow fluorescent protein (YFP) respectively to generate the constructs pV2-YFP^N^ and pV2-YFP^C^. Co-expression of pV2-YFP^N^ and pV2-YFP^C^ by agro-infiltration resulted in a clear YFP fluorescence signal in the cytoplasm of agro-infiltrated cells at 48 hours post-infiltration (hpi). No YFP fluorescence was observed when pV2-YFP^N^ and pYFP^C^ or pYFP^N^ and pV2-YFP^C^ were co-expressed (Fig. 1b), although all constructs were successfully expressed (Fig. 1c). These results demonstrate that V2 specifically interacts with itself in plant cells.

### Biochemical confirmation of the self-interaction of V2

We also demonstrated the self-interaction of TYLCV V2 using a co-immunoprecipitation (Co-IP) assay. In this assay, FLAG-tagged V2 (FLAG-V2) was co-expressed with YFP or YFP-tagged TYLCV V2 (V2-YFP) in *N. benthamiana* by agro-infiltration. Total protein extracts were immunoprecipitated by FLAG-T rap beads (Sigma, USA). The resulting precipitates were analysed by Western blot assays using an anti-YFP antibody (Genscript, USA). We found that V2-YFP was co-immunoprecipitated by FLAG-V2 but YFP was not co-immunoprecipitated by FLAG-V2 (Fig. 1d). In addition, the input recombinant proteins and the loading proteins were both detected successfully using anti-YFP or anti-FLAG antibodies (Sigma, USA). These results reinforce the self-interaction of V2 through a separate methodology and demonstrate that the interaction is independent of the tag.

### Identification the motif of V2 involved in the self-interaction

Through the above experiments, we confirmed that V2 could interact with itself, but determining which domain/motif/sites of V2 were essential for its self-interaction were still unknown.

**Figure 2:**
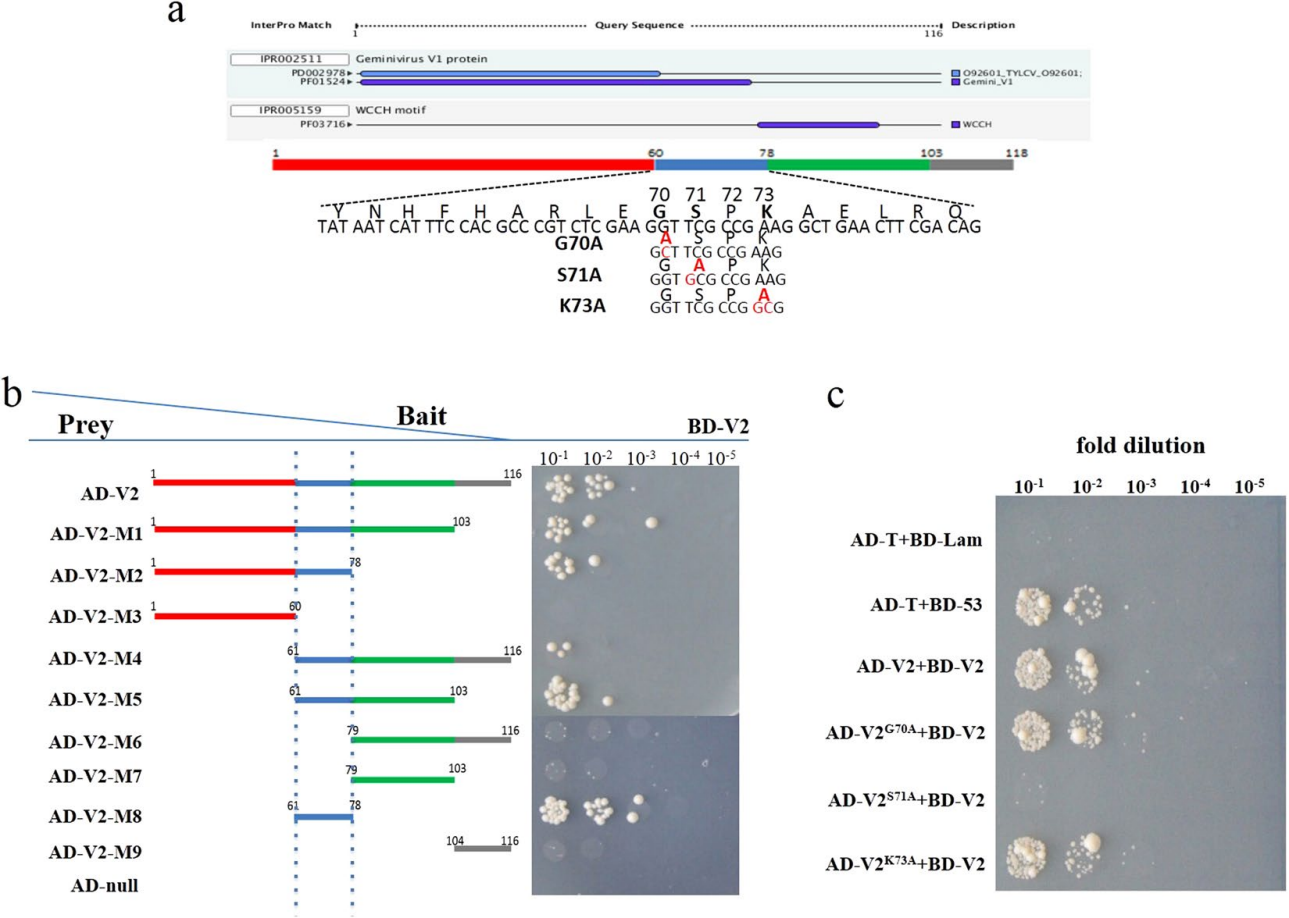
Identification the essential motif and site responsible for the self-interaction of V2. (**a**) Schematic representation of the motif and sites of TYLCV V2 based online prediction. (**b**) Schematic representation of the truncated mutants of V2 and yeast-two-hybrid analysis of the interactions with V2. (**c**) Identification of the interaction between the wide-type V2 and the site-directed mutants V2^G70A^, V2^S71A^, V2^K73A^ in a yeast two-hybrid assay.

To identify the key motif of V2 involved in its self-interaction, nine truncated mutants for V2 were obtained according to the results of online motif scan analysis (http://www.ebi.ac.uk/interpro/) (Fig. 2a), and their effects on the interaction with wild type V2 were tested using yeast two-hybrid assay. As shown in Fig. 2b, yeast transformants harbouring either AD-V2 (amino acids 1–116), AD-V2-M1 (amino acids 1–103), AD-V2-M2 (amino acids 1–78), AD-V2-M4 (amino acids 61–117), AD-V2-M5 (amino acids 61–103) or AD-V2-M8 (amino acids 61–78) together with BD-V2, could grow well on SD/-His/-Leu/-Trp plates, while other mutants for V2 could not grow on the selective medium (Fig. 2b), which indicates that these V2 mutants have lost their ability to interact with wild-type V2. Sequence analysis results revealed that V2-M8 was the necessary region that could interact with wild-type V2 and other mutants that interacted with V2 also contained the motif, which suggests that the motif of amino acids 61 to 78 of V2 are essential for its self-interaction.

### Identification of the sites of V2 involved in the self-interaction

To clarify the approximate sites in the above motif (amino acids 61-78) involved in the self-interaction, three mutants, including V2^G70A^ (the glycine residue at amino acid 70 was changed to alanine), V2^S71A^ (the serine residue at amino acid 71 was changed to alanine), and V2^K73A^ (the lysine residue at amino acid 73 was changed to alanine), were constructed and their effects on the self-interaction were tested.

The yeast two-hybrid assay revealed that transformants containing either AD-V2^G70A^ or AD-V2^K73A^ together with BD-V2 could grow well on selective medium, but transformants containing AD-V2^S71A^ and BD-V2 could not grow on the SD/-His/-Leu/-Trp plates (Fig. 2c), which indicates that S71A mutation of V2 lost its ability to interact with V2. These results showed that the amino acid on position 71 of V2 is a key site for the self-interaction of V2.

The effects of S71A mutation of V2 on its self-interaction was also confirmed by BiFC assay. V2 or V2 mutants were fused to the N- or C-terminal portions of YFP and co-expressed in *N. benthamiana* cells by agro-infiltration. Clear YFP fluorescence signal was observed in the cytoplasm of cells co-infiltrated with pV2-YFP^N^ and pV2-YFP^C^ at 48 hpi, but no fluorescence signal was observed in the cells co-infiltrated with pV2^S71A^-YFP^N^ and pV2-YFP^C^ (Fig. 1b). The result suggested that the amino acid on position 71 is vital for the self-interaction of V2 in plant cells.

The interaction was further confirmed by a co-immunoprecipitation assay. FLAG-tagged V2 and YFP-tagged V2 or V2^S71A^ were co-expressed transiently in *N. benthamiana* by agro-infiltration and subsequently analysed by Western blot assays. V2-YFP was co-immunoprecipitated by FLAG-V2, whereas V2^S71A^-YFP was not found (Fig. 1d), which indicated that V2^S71A^ lost its ability to interact with V2.

All these results confirmed that the S71A mutation of V2 abolish its self-interaction, which indicates the serine on position 71 of V2 is essential for its self-interaction.

### S71A mutation of V2 impaired/interfered with large aggregate formation

**Figure 3:**
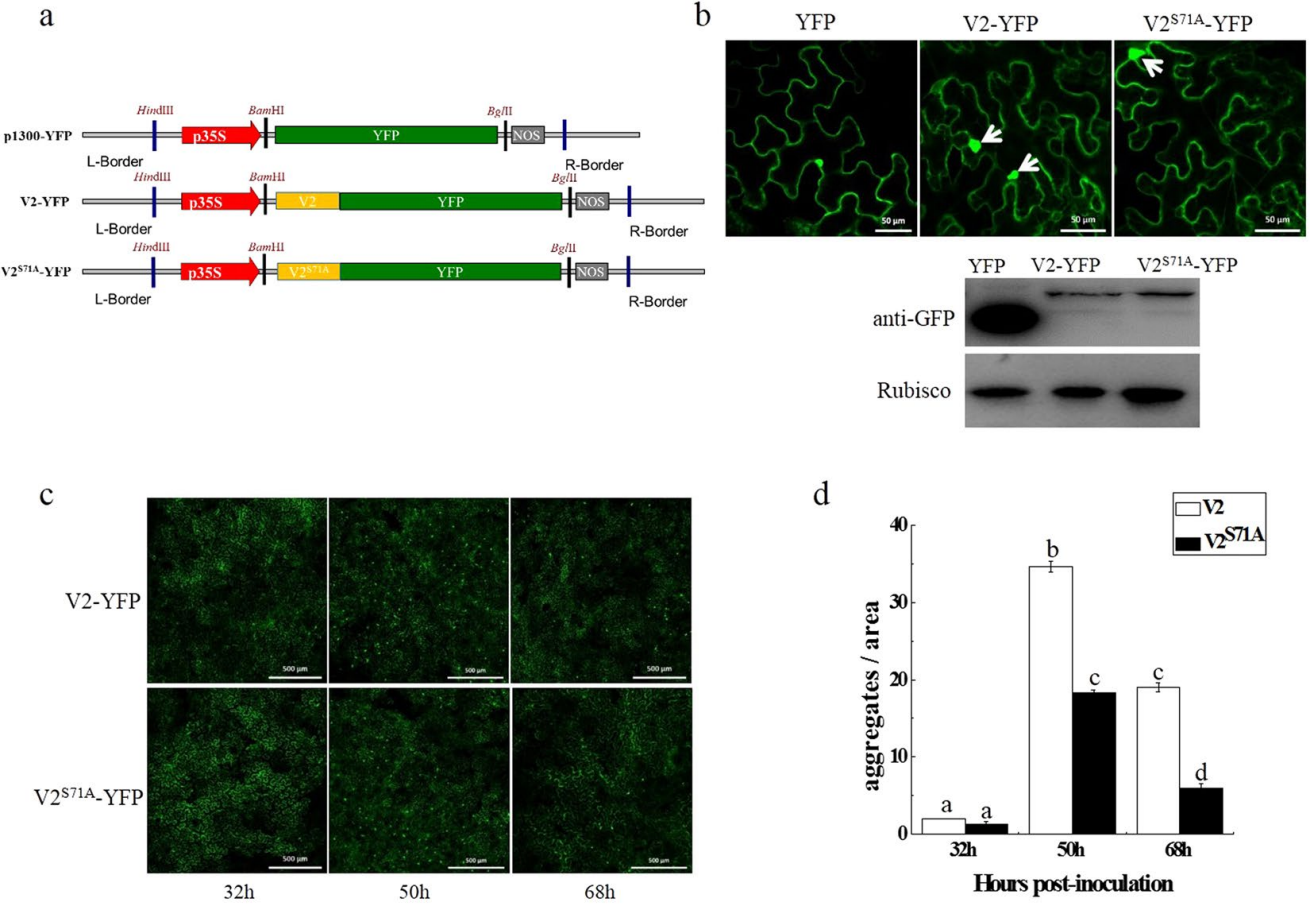
Effects of site-directed mutation of V2 on its large aggregates. (**a**) Diagram of the plant expression vector containing unfused yellow fluorescent protein (YFP) or YFP fused at the N-terminus of V2 and V2^S71A^. L-Bonder is the left border and R-Bonder is the right border of the T-DNA; p35S is the 35S transcript termination signal. (**b**) Expression of V2-YFP and V2^S71A^-YFP in epidermal cells of *N. benthamiana* leaves. The aggregates are indicated with arrows. Bar: 50 μm. Western blot analyses of construct combinations from the same experiment. (**c**) Comparative analysis of large aggregates between V2-YFP and V2^S71A^-YFP in *N. benthamiana* leaves. Bars: 500 μm. (**d**) The statistical number of large aggregates. Bars and numbers indicate the mean number of aggregates as measured for leaf areas of 4 mm^2^ in 10 independent experiments for each treatment. Different lowercase letters above the bars denote significant differences (Fisher’s LSD method; P < 0.05).

V2 and V2^S71A^ tagged with YFP (V2-YFP/V2^S71A^-YFP) were expressed in *N. benthamiana* by agro-infiltration (Fig. 3a). Fuorescence was observed in the cytoplasm and perinucleus regions at 32 hours post-infiltration, which is consistent with previous reports^18^. Compared to V2-YFP, no obvious difference was observed in the subcellular localization of V2^S71A^-YFP (Supplementary Fig. S1).

In addition to cytoplasmic and perinuclear distribution, subcellular localization studies of V2 also revealed that V2 was associated with the formation of large cytoplasmic aggregates. In our study, large aggregates were also observed in V2-YFP and V2^S71A^-YFP (Fig. 3b). Aggregates with increased size were observed in cells infiltrated with V2-YFP or V2^S71A^-YFP, but not with YFP. There was no visible difference at 32 hpi between V2-YFP and V2^S71A^-YFP. However, V2-YFP had 1.5-fold large aggregates than V2^S71A^-YFP at 50 hpi (Fig. 3c,d), which indicates the S71A mutation of V2 has an effect on the formation of large aggregates.

**Figure 4:**
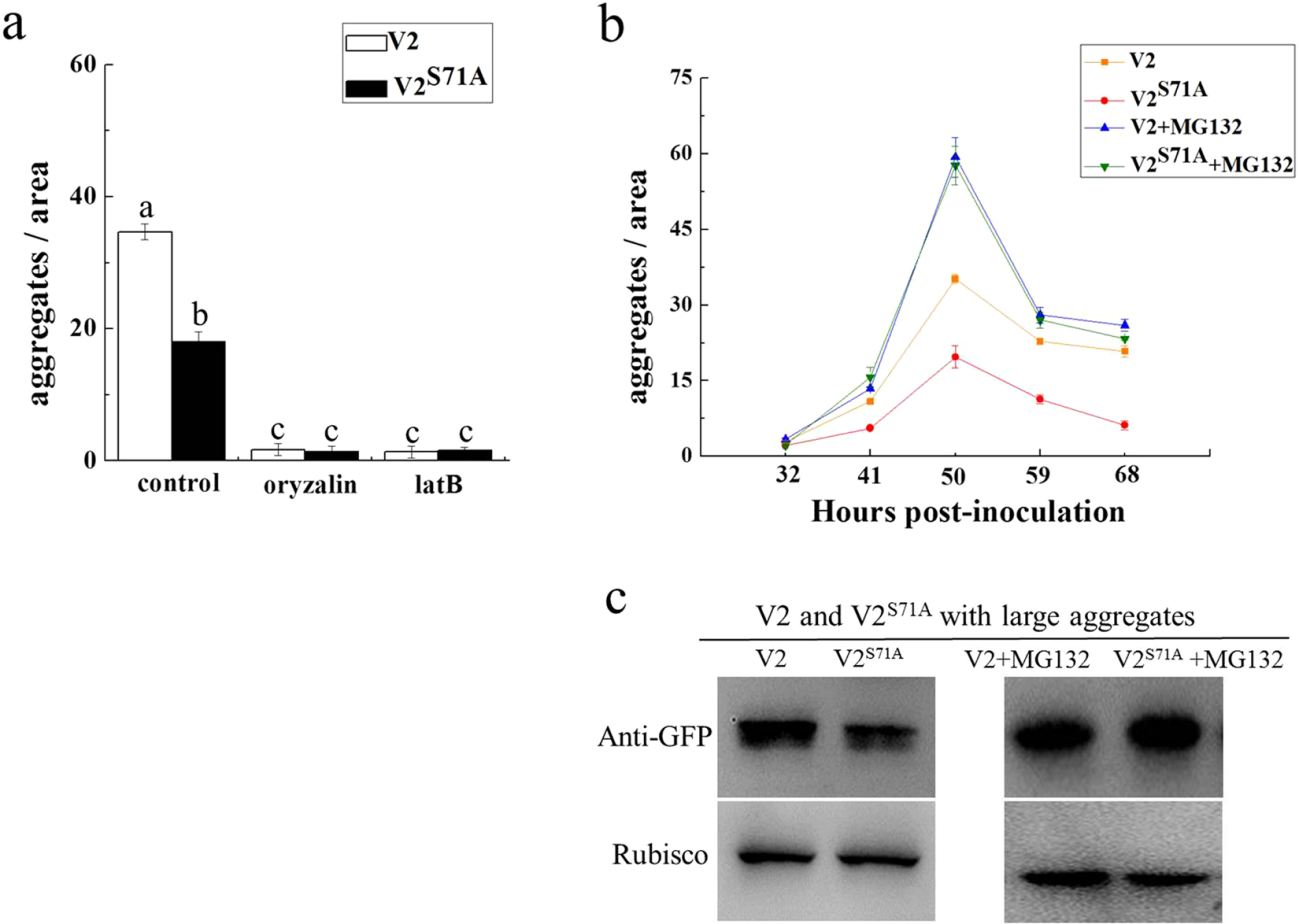
Inhibition of the cytoskeleton and the 26S proteasome lead to changes in the number of V2-YFP and V2^S71A^-YFP large aggregates in *N. benthamiana*. (**a**) Number of large aggregates in latB-treated cells and oryzalin-treated cells. Bars and numbers indicate the mean sizes of aggregates as measured for leaf areas of 4 mm^2^ in 10 independent experiments for each treatment. Different lowercase letters above the bars denote significant differences (Fisher’s LSD method; P < 0.05). (**b**) Number of large aggregates in MG132-treated cells. The average number of aggregates as measured for leaf areas of 4 mm^2^ in 10 independent experiments for each treatment. (**c**) Western blot analysis V2 and V2^S71A^ in large aggregates with or without MG132.

It was reported that the formation of V2 aggregates with increased size and stability depends on the integrity of the actin and microtubule components of the cytoskeleton^18^. Similar to the situation with V2-YFP, large aggregates were rarely observed in oryzalin or latB-treated cells expressing V2^S71A^-YFP (Fig. 4a). These results showed that the disruption of microtubule and actin filaments have similar effects on V2 and V2^S71A^ during the process of large aggregation formation, which indicates that the S71A mutation of V2 has little effect on the cytoskeleton-dependent formation of large aggregates.

The 26S proteasome was also reported to be involved in the degradation of V2 aggregates^18^, and the effects of the S71A mutation of V2 on its 26S proteasome-mediated aggregates degradation were investigated by treating *N. benthamiana* V2-YFP/V2^S71A^-YFP-expressing leaves with the proteasome inhibitor (MG132). Compared to untreated leaves, inhibition of 26S proteasome led to an increase in the number of large aggregates in both V2-YFP and V2^S71A^-YFP infiltrated leaves. The number of large aggregates in V2-YFP and V2^S71A^-YFP infiltrated leaves reached the same level in MG132-treated leaves (Fig. 4b), whereas V2^S71A^-YFP was observed to induce less large aggregates in untreated cells compared to V2-YFP, which indicates there was more large aggregates formation in V2^S71A^-YFP compared V2-YFP after inhibition of the 26S proteasome. We also tested the protein level of V2-YFP and V2^S71A^-YFP in large aggregates, and the result confirmed that there was a decrease in MG132-treated V2^S71A^ samples compared with wide-type V2 (Fig. 4c). Thus, it would appear that the large aggregates of V2^S71A^-YFP seem to be more efficiently degraded by the 26S proteasome system than V2-YFP, which suggests the S71A mutation of V2 is involved in the 26S proteasome-mediated degradation of V2 aggregates and has an effect on their efficiency.

### S71A mutation affects the symptom induction activity of V2

It has been reported that PVX-mediated expression of TYLCV V2 induces severe symptoms in *N. benthamiana*, which suggests V2 is a pathogenicity determinant^23^. To clarify the possible effects of the S71A mutation of V2 on its pathogenicity, recombinant PVX carrying V2 or V2^S71A^ were constructed using the PVX vector pGR107, followed by agro-infiltration in *N. benthamiana*.

**Figure 5:**
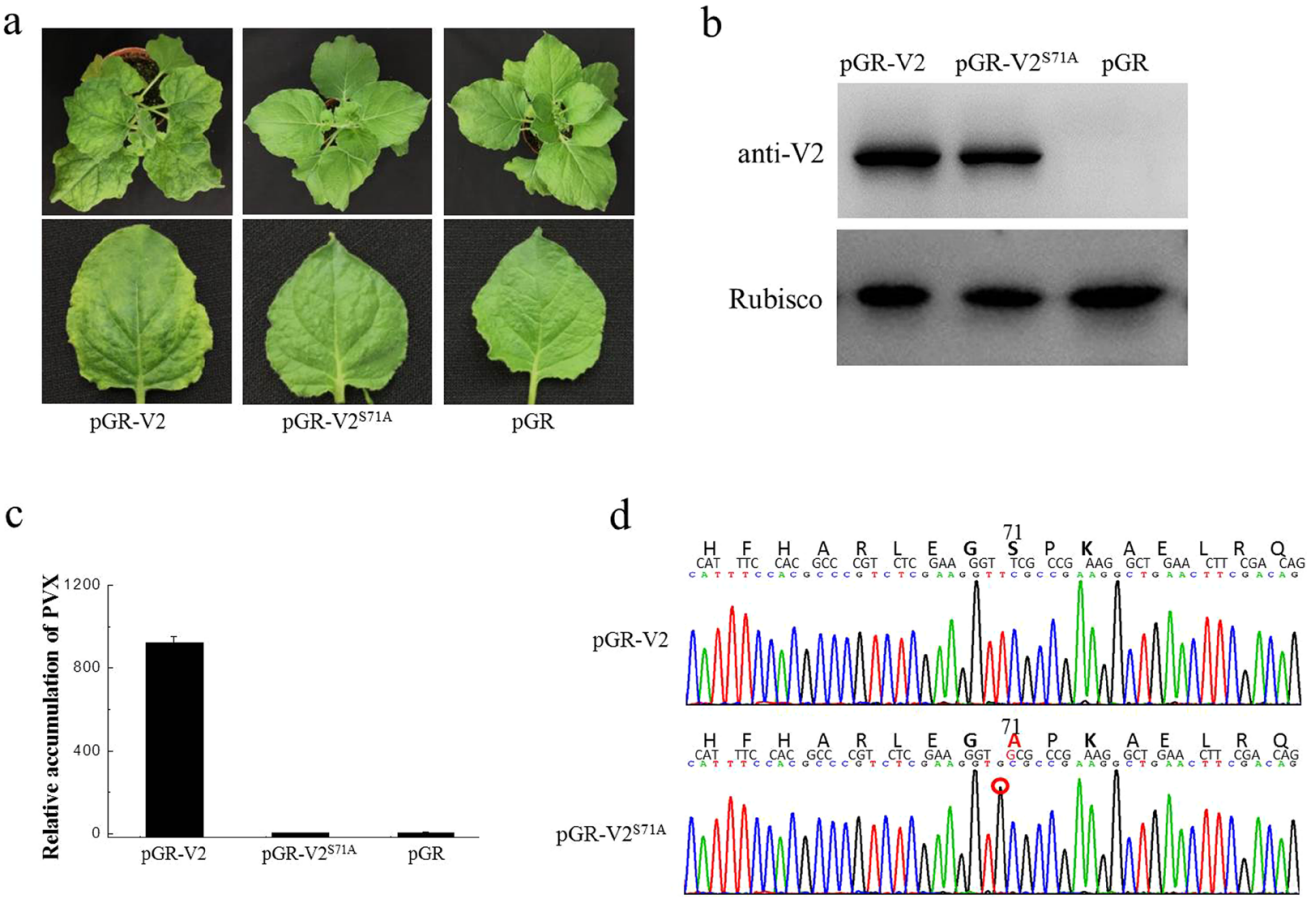
Symptoms exhibited by *N. benthamiana* plants infected with PVX expressing V2 and V2^S71A^. (**a**) The symptoms of *N. benthamiana* plants at 16 dpi. (**b**) Western blot analyses of V2 and V2^S71A^ expression with pGR-V2, pGR-V2^S71A^ and pGR. (**c**) The virus accumulation in plants infiltrated with pGR-V2, pGR-V2^S71A^ and pGR. (**d**) Sequencing analysis of V2 in plants infiltrated with pGR-V2 and pGR-V2^S71A^.

*N. benthamiana* plants infected with pGR-V2 showed leaf curling symptoms as early as 5 dpi, and the symptom severity increased at approximately 8–16 dpi on the newly emerging leaves. Typical symptoms included leaf yellowing and venial necrosis on systemic leaves (Fig. 5a). In contrast, plants infected with pGR-V2^S71A^ only showed mild mosaic symptoms, which was very similar to symptoms induced by the empty PVX vector (Fig. 5a). Real-time PCR results revealed that the accumulation of PVX was significantly increased in the plants inoculated by pGR-V2 (Fig. 5c), whereas no obvious differences were observed in the plants inoculated by pGR-V2^S71A^ or PVX empty vector (Fig. 5c). The presence of V2 and V2^S71A^ was confirmed by Western blot (Fig. 5b) and the mutation of Ser-71 was also confirmed by subsquencent sequencing (Fig. 5d).

These results showed that V2^S71A^ does not induce typical symptoms in plants, and that S71A mutation causes V2 to lose its function as a pathogenicity determinant in the PVX heterogeneous system, which indicates the S71A mutation on V2 is involved in the pathogenicity of V2.

### Effect of S71A mutant on virus infection and viral DNA accumulation

**Figure 6:**
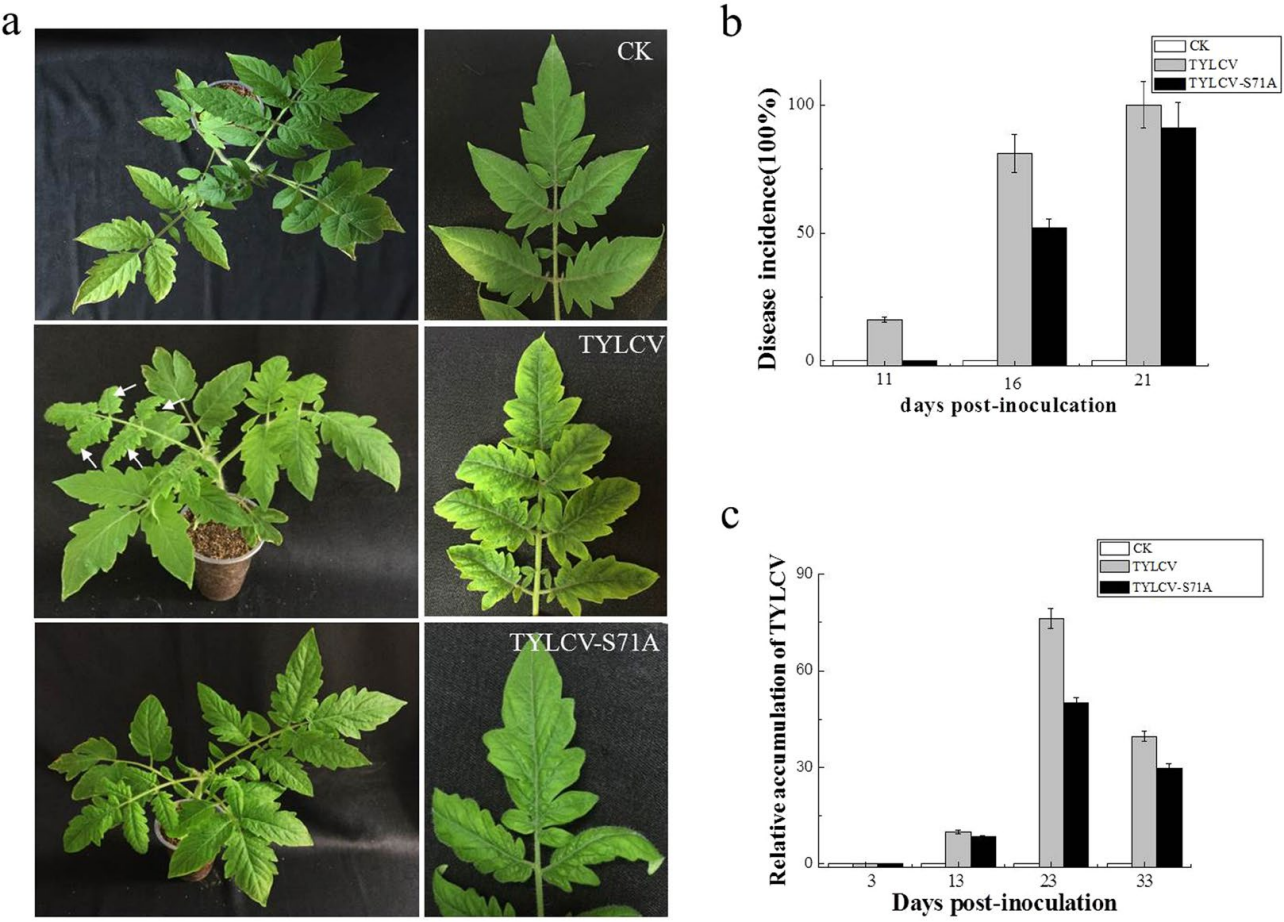
Effects of site mutation S71A on virus infection and relative viral DNA accumulation levels in agroinoculated *solanum lycopersicum* plants. (**a**) Symptoms of plants agroinoculated with TYLCV or TYLCV-S71A at 14 dpi. CK indicates the mock-inoculated plant. (**b**) Infection course of TYLCV or TYLCV-S71A. Values represent percentages of systemically infected plants at different dpi and are given as means ± SD of triplicate experiments. In each experiment, 12 plants were inoculated. (**c**) Accumulation of virus in TYLCV and TYLCV-S71A inoculated plants.

To assess the role of S71A on virus infection, a full length TYLCV mutant (the serine residue on V2 at amino acid 71 was changed to alanine) was generated and the infectious clone(TYLCV-S71A) was constructed. Infectious clone of TYLCV-S71A and wild-type TYLCV were used to inoculate *solanum lycopersicum* plants. Delayed virus infection associated with mild symptoms was observed in plants inoculated with TYLCV-S71A (Fig. 6a,b). Real time PCR showed that viral DNA accumulation was lower in plants inoculated with TYLCV-S71A than in plants inoculated with wild-type TYLCV (Fig. 6c). These data indicated that S71A on V2 caused virus accumulation decreased and reduced the infection effiiciency of TYLCV.

## Discussion

Numerous reports have shown that viral proteins are multifunctional during the processes of infection, replication, movement and pathogenicity^10,29–34^. Many viral proteins interact with viral factors and/or host factors to fulfil the functions required by the virus, including self-interaction. In this study, we found that the TYLCV V2 protein could interact with itself using the yeast two-hybrid system, and the self-interaction was confirmed by bimolecular fluorescence complementation and co-immunoprecipitation. A single amino acid mutation on position 71 of V2 abolished the self-interaction. To the best of our knowledge, this is the first report about self-interaction of V2 encoded by TYLCV.

TYLCV V2 is a well-known viral suppressor of post-transcription gene silencing that counteracts the innate immune response of the host plants, it also inhibits local gene silencing but has no apparent effects on the accumulation of short interfering RNAs^19^. A tomato homologue of the Arabidopsis SGS3 protein (slSGS3) was identified as the host-cell target of V2. The interaction between V2 and slSGS3 was involved in disturbing transportation of the RNA-silencing signal, which suggests there is a correlation between the V2–slSGS3 interaction in plants and the suppressor activity of V2^20^. V2^S71A^ retained the ability to interact with slSGS3 (Supplementary Fig. S2a), which suggests that the mutation S71A might have no effects on the interaction with slSGS3 and the mutation S71A on V2 might have little effect on its suppressor activity.

The host protein CYP1 has also been shown to interact with V2 and V2 down-regulated CYP1 activity to facilitate virus invasion and/or spread^22^. In this study, we also found V2 and V2^S71A^ both interacted with CYP1 (Supplementary Fig. S2b), which indicated that the mutation S71A on V2 might have little effect on its suppression activity of host defence pathways involving CYP1.

It was reported that many viruses induce the formation of aggregates/inclusion bodies inside infected cells, which usually contained viral and host proteins and varying in location, size, content and biological function, but less is known about the role of the aggregation of viral proteins during infection in plant cells. The V2 protein was found to be associated with large cytoplasmic aggregates, and the formation of V2 aggregates were associated with the integrity of the actin and microtubule cytoskeleton as well as the 26 S proteasome-mediated degradation, which was consistent with the Moshe’ report^18^. Large cytoplasmic aggregate bodies were also found in V2^S71A^, although the number of aggregates was smaller compared to the wild-type V2 protein. When treated with oryzalin or latrunculin B, the number of large aggregates diminished, which was similar to the effects of wild-type V2, which suggests mutation S71A on V2 might have little effect on the actin filaments and microtubule dependent aggregate formation. When treated with MG132, the number of large cytoplasmic aggregates of V2^S71A^ increased and showed similar levels compared to wild-type V2, which indicats that mutation S71A on V2 might affect 26S proteasome-mediated degradation of the large cytoplasmic aggregates.

It was reported that viral proteins of TYLCV in the intermediate-sized aggregates were less exposed to degradation compared with large aggregates, which proposed TYLCV could confront host degradation by sheltering in small/midsized aggregates, where viral proteins are less exposed to proteolysis, except large aggregates^35^. As V2^S71A^ had less large aggregates compared to wide-type V2, and the serine on position 71 of V2 is essential for self-interaction, which proposed that the self-interaction of V2 might be involved in a counter-defense response for TYLCV to protect the large aggregates against host pant 26S proteasome mediated degradation.

We tested the pathogenicity of V2 using a PVX expression system. *N. benthamiana* plants infiltrated with pGR-V2 showed severe symptoms, including severe leaf yellowing and venial necrosis on systemic leaves at 16 dpi (Fig. 5a), which indicates V2 is a pathogenicity determinant in the PVX heterogeneous system and that outcome is consistent with previous reports^23^. No severe symptoms but only mild mosaic symptoms were observed on *N. benthamiana* plants infiltrated with pGR-V2^S71A^ until 21 dpi, which was similar to the symptoms induced by the empty PVX vector pGR107. These results indicate that the serine on position 71 is essential for the pathogenicity of V2, which suggests there might be a correlation between the self-interaction of V2 and the pathogenicity of V2. The infectious clone of wild type TYLCV and TYLCV-S71A were also constructed and the effects of S71A to virus infection was tested. The result revealed that the S71A mutation on V2 did has effects on virus infection, causing lower viral DNA accumulation and mild symptoms.

Multimerisation of viral proteins play an important role in the life cycle of virus^36,37^. In *Cucumovirus*, the self-interaction of the 2b protein encoded by *Cucumber Mosaic Virus* (CMV) and *Tomato Aspermy Virus* (TAV) was demonstrated using BiFC, which proposed that 2b functions as a suppressor of RNA silencing in the form of a homodimer or higher order oligomer^38–40^. In *Fijivirus*, the self-interaction and high-order oligomers of P9-1 protein encoded by *Rice black-streaked dwarf virus* (RBSDV) were also found to be involved in the formation of viroplasm^41,42^. In *Begomovirus*, it was reported that the AL2 protein encoded by *Tomato golden mosaic virus* (TGMV) could interact with itself and form different oligomers with different subcellular localization and functions. Multimeric structures of AL2 were located in the nucleus to activate transcription, whereas monomers were located in the cytoplasm to suppress local silencing^27^. In this study, TYLCV V2 was demonstrated that it could interact with itself, which prompted us to investigate whether V2 was capable of forming multimeric structures and what were its biological functions. There was no evidence to support the presence of multimeric complexes of V2 was affected by the self-interaction or not yet, so further research on V2 multimerisation and associated biological functions should be addressed.

Here, we investigated the self-interaction of V2 and found mutation of S71A on V2 abolished its self-interaction and affect V2 large aggregates formation and pathogenicity. The serine on position 71 of V2 is a potential phosphorylation site based on an online prediction. It was reported that the Ser188 phosphorylation of RhoA, a major regulator of the actin cytoskeleton, by cAMP and cyclic GMP-dependent kinases (PKA and PKG) could protect RhoA, particularly its active form, from ubiquitin-mediated proteasomal degradation, regulating RhoA expression and functions in vascular smooth muscle cells. The recent research on tyrosine hydroxylase (TH), a rate-limiting enzyme in catecholamine biosynthesis, also revealed that the phosphorylation of TH at its Ser19 also had effects on its proteasome-mediated degradation^43,44^. Whether there is a correlation between the phosphorylation on the serine on position 71 and the functions of V2 should be investigated in future research.

## Materials and Methods

### Plant Materials and Growth Conditions

All agro-infiltration experiments were performed in *Nicotiana benthamiana*. The plants were grown in a growth chamber (ModelGXZ500D, Jiangnan Motor Factory, Ningbo, China) with temperatures ranging from 26 °C (16 h, light) to 22 °C (8 h, dark) for 1–1.5 months before infection with the viruses. After infiltration, the plants were kept under the same growth conditions.

### Plasmid Construction

The coding sequence for the TYLCV V2 gene was amplified from the cDNA of a TYLCV-infected tomato plant from Jiangsu Province, China (GenBank accession number GU111505)^45^ using corresponding primers (Supplementary Table S1).

Site-directed mutants V2^G70A^, V2^S71A^, V2^K73A^ were synthesized (Invitrogen, China) based on the sequence of wide-type of V2 and confirmed by sequencing (Fig. 2a).

To prepare plasmids for the yeast two-hybrid system, the full-length coding sequence of V2 (residues 1–116), truncated mutants of V2-M1 (residues 1–103), V2-M2 (residues 1–78), V2-M3 (residues 1–60), V2-M4 (residues 61–116), V2-M5 (residues 61–103), V2-M6 (residues 79–116), V2-M7 (residues 79–103), V2-M8 (residues 61–78), V2-M9 (residues 104–116), and site-directed mutants V2^G70A^, V2^S71A^, V2^K73A^ were PCR-amplified with corresponding primer couples shown in Supplementary Table S1. The amplified products were then cloned into the pGBKT7 or pGADT7 vector (Clontech, Mountain View, CA, USA) at the *Eco*RI/*Nde*I digestion sites to produce the recombinant plasmids BD-V2 and AD-V2 as well as AD-V2-M1, AD-V2-M2, AD-V2-M3, AD-V2-M4, AD-V2-M5, AD-V2-M6, AD-V2-M7, AD-V2-M8, AD-V2-M9, AD-V2^G70A^, AD-V2^S71A^ and AD-V2^K73A^ respectively.

To investigate the subcellular localization, the TYLCV V2 and V2^S71A^ genes (*Bgl*II) were amplified using specific primers for PCR (Supplementary Table S1). The yellow fluorescent protein (YFP) gene was inserted between the 35 S promoter of CaMV and the 35 S terminator (35 St) in the pCambia1300 binary vector through standard molecular cloning procedures to obtain p1300-YFP. Then corresponding cDNA was inserted into the *Bam*HI (compatible with *Bgl*II) site of p1300-YFP vector and fused in frame with YFP at the N-terminus to generate V2-YFP and V2^S71A^-YFP.

For the production of BiFC vectors, the full-length coding sequence of V2 and V2^S71A^ gene (*Bgl*II) were amplified using the primers listed in Supplemental Table S1 and cloned into the *Bam*HI site as a fusion with the N-terminal fragment of YFP, resulting in pV2-YFP^N^ and pV2^S71A^-YFP^N^. The full-length coding sequence of V2 was cloned into the C-terminal fragment of YFP, resulting in pV2-YFP^C^.

FLAG tagged V2 (*Bgl*II) was amplified using specific primers listed in Supplementary Table S1 and inserted the *Bam*HI site between the 35S promoter and the 35St in the pCambia1300 binary vector to generate FLAG-V2 for the future Co-IP experiment.

To test whether V2 acts as a determinant of TYLCV pathogenicity, the TYLCV V2 gene was amplified using specific primers in PCR (Supplementary Table S1). The resulting construct was digested by *Cla*I and *Sal*I enzymes and inserted into pGR107^46^.

For the construction of infectious clones of TYLCV containg V2^S71A^, a full length TYLCV mutant, TYLCV-S71A (the serine residue on V2 at amino acid 71 was changed to alanine), was generated. Then the full DNA-A of TYLCV-S71A was amplified using primers listed in Supplemental Table S1 and inserted into the pGEM-T Easy (Promega) vector to produce pGEM-1A. After sequence confirmation, a 2183 nucleotide (nt) fragment was excised from pGEM-1A with *Bam*HI and *Sac*I, then subcloned into the *Bam*HI-*Sac*I sites of the binary vector pBinPLUS to produce pBinPLUS-0.8A. The full-length of TYLCV-S71A was digested from pGEMT-1A with *Bam*HI and inserted into pBinPLUS-0.8A at its unique *Bam*HI site to yield clone pBinPLUS-1.8A. The infectious clone of wide-type TYLCV was constructed following similar procedure.

### Subcellular Localization of Proteins

P1300-YFP, V2-YFP and V2^S71A^-YFP were introduced, respectively, into *A. tumefaciens* strain GV3101 through electroporation. Leaves of 4-week-old *N. benthamiana* plants were infiltrated with *A. tumefaciens* harbouring the described constructs. After 32 hpi, leaf explants were excised and YFP fluorescence was examined in epidermal cells using confocal microscopy (ZEIZZ LSM 710). The microscope was configured with a 458–515 nm dichroic mirror for dual excitation and a 488-nm beam splitter to help separate YFP fluorescence. The number of larger aggregates (>20 μm^2^) per image-area (4 mm^2^) were measured in 10 independent experiments.

### Inhibitor Treatments

Oryzalin, latrunculin B (latB) (Sigma, USA) and MG132 (Calbiochem, Germany) were dissolved in dimethyl sulfoxide (DMSO) to prepare 100 mM stock solutions. For *in vivo* treatment of *N. benthamiana* leaves, stock solutions were diluted in water to prepare solutions of 20 μM oryzalin, 10 μM latB and 50 μM MG132. The freshly prepared solutions were put in a syringe and used to treat the abaxial leaf side 24 h after agro-infiltration of V2-YFP or V2^S71A^-YFP. The leaves were examined under a microscope 15–20 h after inhibitor infiltration. Control infiltrations were performed using the same final concentration of DMSO without the drug.

### Yeast Two-Hybrid Assay

The yeast two-hybrid system was used to identify interactions between TYLCV V2 with itself, a truncated mutant or site-directed mutant.

V2 and V2 truncated mutants (V2-M1, V2-M2, V2-M3, V2-M4, V2-M5, V2-M6, V2-M7, V2-M8, V2-M9) and site-directed substitution mutants (V2^G70A^, V2^S71A^, V2^K73A^) were linked to the B42 activation domain (AD)-containing vector expression vector then co-transformed into *Saccharomyces cerevisiae* AH109 with the LexA DNA binding domain (BD) containing V2 (BD-V2) to identify interactions. The plasmids BD-53 and AD-recT served as a positive control, plasmids BD-Lam and AD-recT were used as a negative control. Transformants grew at 30 °C for 72 h on SD/-His/-Leu/-Trp/synthetic medium and/or then transferred to the SD/-Ade/-His/-Leu/-Trp/medium containing X-α-gal to identify binding activity. Three independent experiments were performed to confirm the results.

### Bimolecular Fluorescence Complementation (BiFC) Assay

BiFC experiments were performed as described previously^47^. pV2-YFP^N^, pV2^S71A^-YFP^N^ and pV2-YFP^C^ were introduced individually into *A. tumefaciens* strain GV3101 by electroporation. Agro-infiltration was performed with an overnight culture of the *A. tumefaciens* strain GV3101 carrying pV2-YFP^N^ and pV2-YFP^C^ or pV2^S71A^-YFP^N^ and pV2-YFP^C^. After agro-infiltration, *N. benthamiana* plants grew in a growth chamber with a 16 h light/8 h dark.

YFP fluorescence was observed and photographed through confocal microscopy (ZEIZZ LSM 710) at 48 h after infiltration. YFP was observed under a mercury lamp light using a 488-nm excitation filter. Photographic images were prepared by using ZEN 2011SP1. Three independent experiments were performed to confirm the results.

### Co-Immunoprecipitation

*A. tumefaciens* strain GV3101 carrying V2-YFP, V2^S71A^-YFP, FLAG-V2 and V2-YFP, FLAG-V2 and V2^S71A^-YFP or FLAG-V2 and p1300-YFP were co-infiltrated in *N. benthamiana* plants as descripted in Fig. 1d.

40 hours after infiltration, the tobacco leaves were harvested and ground in liquid nitrogen. Proteins were extracted in extraction IP buffer (40 mM Tris-HCl at pH 7.5, 100 mM NaCl, 5 mM MgCl_2_, 2 mM EDTA, 2x EDTA-free proteinase inhibitor, 1 mM PMSF, 4 mM DTT, 1% glycerol, and 0.5% Triton-X100).

Next, 0.4 g of each samples were ground into powder and the powder was mixed with 0.8 ml extraction buffer. The cell debris was pelleted by centrifugation at 12,000 g for 15 min. The supernatant was removed to incubate the samples with FLAG-conjugated beads. After 1 h of incubation at 4 °C, the beads were centrifuged and washed six times with IP buffer. Proteins were eluted by 20 µL of 2x SDS sample buffer and analysed by Western blot using a monoclonal anti-YFP antibody to survey whether V2-YFP can combine with FLAG-V2 or not. Meanwhile, after collecting the input sample and the loading proteins with 2x SDS buffer, both proteins were successfully detected using relative anti-YFP or anti-FLAG antibodies analysed with Western blot. Three independent experiments were performed to confirm the results.

## Electronic supplementary material

Supplementary information
