# Supplementary material for: Single amino acid in V2 encoded by TYLCV is responsible for its self-interaction, aggregates and pathogenicity
Source: Sci Rep. 2018 Feb 23;8:3561. doi: 10.1038/s41598-018-21446-2 (PMC5824789; doi:10.1038/s41598-018-21446-2)
Supplement: Supplementary file 1 — Supplementary information [file 41598_2018_21446_MOESM1_ESM.pdf]

Single amino acid in V2 encoded by TYLCV is responsible for its self-interaction, aggregates and pathogenicity

Wenhao Zhao[#1, 2], Yinghua Ji[#2,1], Shuhua Wu[2], Xiaofang Ma[2], Shuo Li[2], Feng Sun[2], Zhaobang Cheng[2], Yijun Zhou[2]*, and Yongjian Fan[1, 2]*

Supplementary information

Supplementary Table S1

	Designation	Sequence(5' to 3')	Assay
1	TY-V2-F-AB	CGCCATATGATGTGGGATCCACTTCTAAAT	AD-V2,<br>BD-V2,<br>AD-V2-M1,<br>AD-V2-M2,<br>AD-V2-M3,<br>AD-V2^{G70A},<br>AD-V2^{S71A},<br>AD-V2^{K73A}
2	TY-V2-R-AB	CGGAATTCTCAGGGCTTCGATACATTCT	AD-V2,<br>BD-V2,<br>AD-V2-M4,<br>AD-V2-M6,<br>AD-V2-M9,<br>AD-V2^{G70A},<br>AD-V2^{S71A},<br>AD-V2^{K73A}
3	TY-V2-R-AB-180	CGGAATTCTCGCCTGGTCGCTTCGAC	AD-V2-M3
4	TY-V2-R-AB-234	CGGAATTCCTGTCGAAGTTCAGCCTTC	AD-V2-M2,<br>AD-V2-M8
5	TY-V2-R-AB-309	CGGAATTCATGGGCCTGTACGTCCAT	AD-V2-M1,<br>AD-V2-M5,<br>AD-V2-M7
6	TY-V2-F-AB-170	CGCCATATGTATAATCATTTCCACGCCCG	AD-V2-M4,<br>AD-V2-M5,<br>AD-V2-M8
7	TY-V2-F-AB-116	CGCCATATGCCCATACAGCAGCCGTGC	AD-V2-M6,<br>AD-V2-M7
8	TY-V2-F-AB-42	CGCCATATGGTACCGGAAGCCCAGAATA	AD-V2-M9
9	V2-bgF	GAAGATCTATGTGGGATCCACTTCTAAAT	V2-YFP,<br>pV2-YFP^{N},<br>pV2-YFP^{C},<br>V2^{S71A}-YFP,<br>pV2^{S71A}-YFP^{N}

10	V2-bgR	GAAGATCTGGGCTTCGATACATTCTGTAT	V2-YFP, pV2-YFP^{N}, pV2-YFP^{C}, V2^{S71A}-YFP, pV2^{S71A}-YFP^{N}, FLAG-V2
11	TY-V2-F-Cla	CCATCGATTGTGGGATCCACTTCTAAAT	pGR-V2, pGR- V2^{S71A}
12	TY-V2-R	GCGTCGACTCAGGGCTTCGATACATTCT	pGR-V2, pGR- V2^{S71A}
13	FLAG-V2-F	GAAGATCTATGGATTACAAGGATGATGATGAT AAGTGGGATCCACTTCTAAATG	FLAG-V2
14	TY-1A-F	GGGGTACCACTTCTAAATGAATTTCCTGAATC TG	1A, 1A-S71A
15	TY-1A-R	CGGGATCCCACATAGTGCAAGACAAACT	1A, 1A-S71A

Supplementary Figure S1 Subcellular localization of V2 and V2^{S71A}. Bars: 50 μm.

Supplementary Figure S2 Effects of mutants on the interactions of V2 with slSGS3 and CYP1. (a) Co-expressing V2 or V2^{S71A} and slSGS3, yeast transformants can grow on an SD/-His/-Leu/-Trp medium. (b) Co-expressing V2 or V2^{S71A} and slCYP1, yeast transformants can grow on an SD/-His/-Leu/-Trp medium.

Supplementary Figure S1

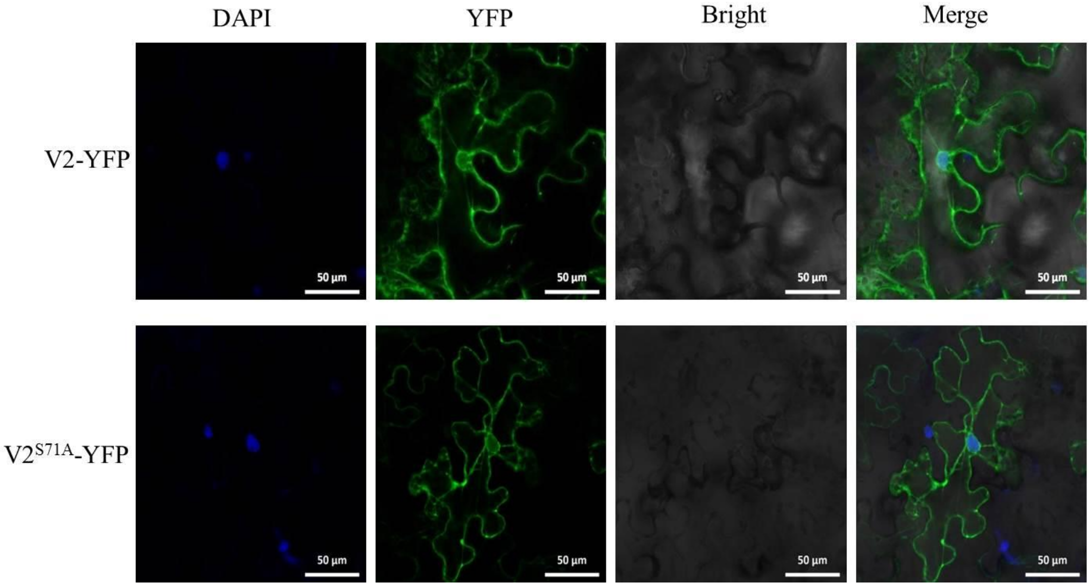

Supplementary Figure S2

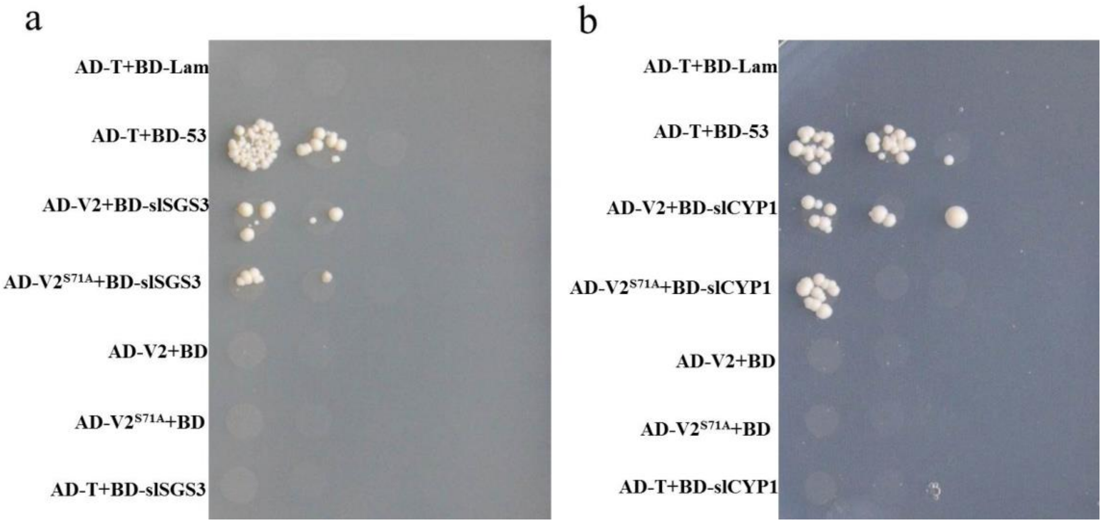
a
AD-T+BD-Lam
AD-T+BD-53
AD-V2+BD-slSGS3
AD-V2S71A+BD-slSGS3
AD-V2+BD
AD-V2S71A+BD
AD-T+BD-slSGS3
b
AD-T+BD-Lam
AD-T+BD-53
AD-V2+BD-slCYP1
AD-V2S71A+BD-slCYP1
AD-V2+BD
AD-V2S71A+BD
AD-T+BD-slCYP1
